# Identification of microvascular and morphological alterations in eyes with central retinal non-perfusion

**DOI:** 10.1371/journal.pone.0241753

**Published:** 2020-11-10

**Authors:** Dorottya Hajdu, Reinhard Told, Orsolya Angeli, Guenther Weigert, Andreas Pollreisz, Ursula Schmidt-Erfurth, Stefan Sacu

**Affiliations:** 1 Department of Ophthalmology and Optometry, Vienna Clinical Trial Centre (VTC), Medical University of Vienna, Vienna, Austria; 2 Department of Ophthalmology, Semmelweis University, Budapest, Hungary; 3 Christian Doppler Laboratory for Ophthalmic Image Analysis, Vienna Reading Center, Department of Ophthalmology and Optometry, Medical University of Vienna, Vienna, Austria; Nicolaus Copernicus University, POLAND

## Abstract

**Purpose:**

To evaluate the characteristics and morphological alterations in central retinal ischemia caused by diabetic retinopathy (DR) or retinal vein occlusion (RVO) as seen in optical coherence tomography angiography (OCTA) and their relationship to visual acuity.

**Methods:**

Swept-source optical coherence tomography (SSOCT) and OCTA (Topcon, Triton) data of patients with central involving retinal ischemia were analyzed in this cross-sectional study. The following parameters were evaluated: vessel parameters, foveal avascular zone (FAZ), intraretinal cysts (IRC), microaneurysms (MA), vascular collaterals in the superficial (SCP) and deep plexuses (DCP), hyperreflective foci (HRF), epiretinal membrane (ERM), external limiting membrane (ELM) and ellipsoid zone (EZ) disruption, as well as the disorganization of retinal inner layers (DRIL). Best-corrected visual acuity (BCVA), age, gender, disease duration and ocular history were also recorded.

**Results:**

44 eyes of 44 patients (22 with RVO, 22 with DR) were analyzed. The mean age was 60.55 ± 11.38 years and mean BCVA 0.86 ± 0.36 (Snellen, 6m). No significant difference was found between DR subgroups (non proliferative vs. proliferative). Between RVO subgroups (CRVO vs. BRVO) a significant difference was found in term of collateral vessel of the DCP (p = 0.014). A pooled DR and RVO group were created and compared. Significantly more MAs (p = 0.007) and ERM (p = 0.007) were found in the DR group. Statistically significant negative correlation was demonstrated between FAZ and BCVA (p = 0.45) when analyzing all patients with retinal ischemia.

**Conclusion:**

This study has shown that the best predictor of visual outcome in center involved ischemic diseases is the size of FAZ. Besides the presence of MAs and ERM, all other OCT and OCTA parameters were present in a similar extent in DR and RVO group despite the completely different disease origins. Our results suggest that as soon as retinal ischemia in the macular region is present, it has a similar appearance and visual outcome independently of the underlying disease.

## Introduction

Retinal ischemia is a common ophthalmological pathology with many different complications that can cause visual impairment and may eventually lead to blindness. Ischemia is a pathological condition involving inadequate blood flow and inability to satisfy cellular energy demands [1].

In retinal diseases with chronic vascular obstructions, it is difficult to predict the development of ischemia and the functional outcome [1]. Ganglion cell loss in the ischemic areas was clearly demonstrated in previous studies and it has also been observed that the outer retinal layers are more sensitive to nonperfusion. Over time, most layers of the retina become thinner and eventually even photoreceptor degeneration can occur [1, 2]. Several optical coherence tomography (OCT) features such as external limiting membrane (ELM) disruption, ellipsoid zone (EZ) disruption, hyperreflective foci (HRF) and disorganization of retinal inner layers (DRIL) were described as predictors of retinal ischemia. Also, they were found to be associated with visual acuity [3]. Consistent relationship between foveal avascular zone (FAZ) enlargement and visual function decrease was shown in multiple studies based on patients with DR and RVO [4, 5].

In fact, DR and RVO are the two most common retinal microangiopathies with completely different disease origins [1]. In diabetic patients high blood sugar, increased oxidative stress, inflammation and hypoxia lead to basement membrane disorder, vessel thickening, loss of pericytes and degenerative changes in the ganglion cell and nerve fiber layer [6]. Consequently, diabetic retinopathy develops vascular changes over time which include occlusion of small capillaries causing non-perfused areas [6].

The risk factors for RVO are systemic diseases including hypertension, hyperlipidemia, diabetes mellitus and arteriosclerosis. Patients with hematological or inflammatory diseases are also affected. RVO is grouped based on the occluded vascular segment, which comprise central retinal vein occlusions (CRVO) and branch retinal vein occlusion (BRVO). On the basis of nonperfusion, ischemic and non-ischemic venous occlusion can be distinguished [1]. The resulting retinal ischemia may lead to considerable vision loss and further complications, such as macular edema and retinal neovascularization.

With the increasing availability of OCT angiography (OCTA), it has become possible to visualize the retinal and choroidal vasculatures. Besides the aforementioned structural changes observed in OCT, OCTA may give additional insight into the vascular changes of retinal ischemia. We used OCTA parameters, such as vessel density (VD), junction density, vessel length, number of endpoints and lacunarity as these provide information on the central perfusion status and allow to estimate the extent of the central retinal ischemia [5, 7].

Hence, the aim of this cross-sectional observation study was to analyze OCT and OCTA data from patients with retinal ischemia caused by different diseases (DM, RVO). The morphological alterations associated with central retinal non-perfusion as well as their association with visual acuity were also investigated.

## Methods

The present study was performed in adherence to the Declaration of Helsinki including current revisions and the Good Clinical Practice (GCP) guidelines. The study protocol was approved by the Ethics Committee of the Medical University of Vienna. In this retrospective, cross-sectional study, we included data acquired at the Department of Ophthalmology and Optometry of the Medical University of Vienna, Austria from October 2016 to June 2019. The data were reviewed for potential study participants on the basis of the specific inclusion and exclusion criteria. All participants were numbered sequentially and pseudonymized for further evaluation. Patient names were kept secure in the investigator file, as required and approved by the Ethics Committee. Inclusion criteria for this study were the following: (1) center involving retinal ischemia on OCTA (2) diagnosis of DR or RVO (3) no clinically significant macular edema (4) clear ocular media and good image quality (5) absence of other concurrent ocular diseases (6) patients receiving intravitreal anti-VEGF treatment during 8 weeks before potential study inclusion, were not included in this study. Peripheral laser treatment was allowed, however, patients with steroid injections, implants or central laser treatment were not enrolled. Patients who met the criteria were selected by the same investigator based on OCTA images. To determine retinal ischemia, OCTA images were first evaluated subjectively. Images with at least one third nonperfusion of the whole 6x6mm image were defined as having a center involving ischemia and were included in the analysis. We clarified peripheral ischemia of the patients from wide-field fluorescein angiographies to create two comparable cohorts with similar peripheral nonperfusion status. Wide-field angiography (120°) was analyzed using Heidelberg Spectralis (Heidelberg Engineering, Heidelberg, Germany) from the RVO patients. Ultrawide field (200°) angiographies were routinely performed of DR patients using Optos California. (Optos, Optomap, North America). To make these images comparable we only graded the same field of view (approximately 120°) of these Optomap images. Three groups were created to quantify peripheral ischemia based on the extension of nonperfusion: no, ≤5 disc diameter or >5 disc diameter peripheral ischemia. The results of these analyses were not included in the statistical evaluation.

The affected eye of RVO patients and randomly one eye of the diabetic patients was selected. Diabetic retinopathy classification was based on clinical examination using the early treatment diabetic retinopathy study (ETDRS) criteria [8]. Best-corrected visual acuity (BCVA) was also documented (Snellen chart, 6m).

### Swept-source optical coherence tomography angiography (SSOCTA)

All patients were examined after pupil dilatation with Tropicamide (Mydriaticum Agepha, Austria) using the Topcon DRI-OCT Triton swept-source OCT/-A (Topcon, Japan). The SSOCTA device uses a 1050-nm swept-source with an A-scan rate of 100.000. The SSOCTA device operates with the OCTARA (OCTA ratio analysis) algorithm, which provides improved detection sensitivity of low blood flow and reduces motion artifacts without compromising axial resolution [9]. A 6x6-mm volumetric flow-scan centered on the fovea was recorded for each eye. Exported enface images had a resolution of 320 x 320 pixels. The automated layer segmentation displayed the predefined vascular plexuses. These were the superficial and deep retinal capillary plexus, the outer retina to choriocapillaris and the choroidal capillary layer in orthogonal view. The settings for layer segmentation of the Topcon software were the following:

1. superficial plexus from internal limiting membrane (ILM) + 2.6 μm to inner plexiform/ inner nuclear layer (IPL/INL) + 15.6 μm,
2. deep capillary plexus from IPL/INL + 15.6 μm to IPL/INL+ 70.2 μm,
3. outer retina from IPL/INL + 70.2 μm to Bruch’s membrane (BM) and
4. choriocapillaris from BM to BM+ 10.4 μm.

Corrections of the layer segmentation whenever necessary were made manually. The corresponding structural B-scans were used to guide placement of the segmentation lines.

### SSOCTA variables

**Fig 1:**
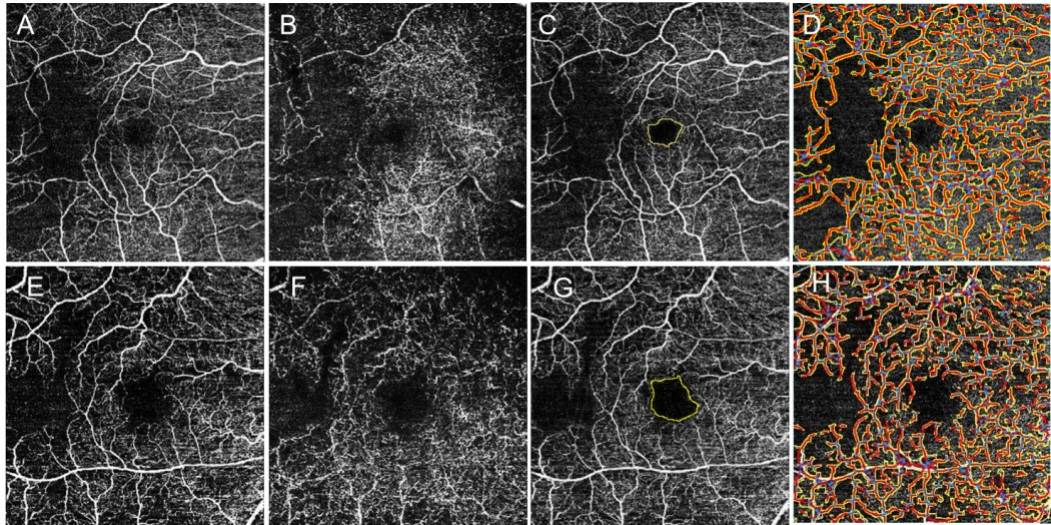
OCTA images of patients with pronounced ischemia. A 52-year-old patient with severe DR above (**A-D**), a 57-years-old male patient with CRVO below (**E-H**). (**A**) superficial capillary plexus of the diabetic patient; (**B**) deep capillary plexus of the diabetic patient; (**C**) yellow line indicates the FAZ area of the diabetic patient; (**D**) AngioTool analysis of the superficial plexus of the diabetic patient; (**E**) superficial capillary plexus of the RVO patient; (**F**) deep capillary plexus of the RVO patient; (**G**) yellow line indicates the FAZ area of the RVO patient; and (**H**) AngioTool analysis of the superficial plexus of the RVO patient.

**Fig 2:**
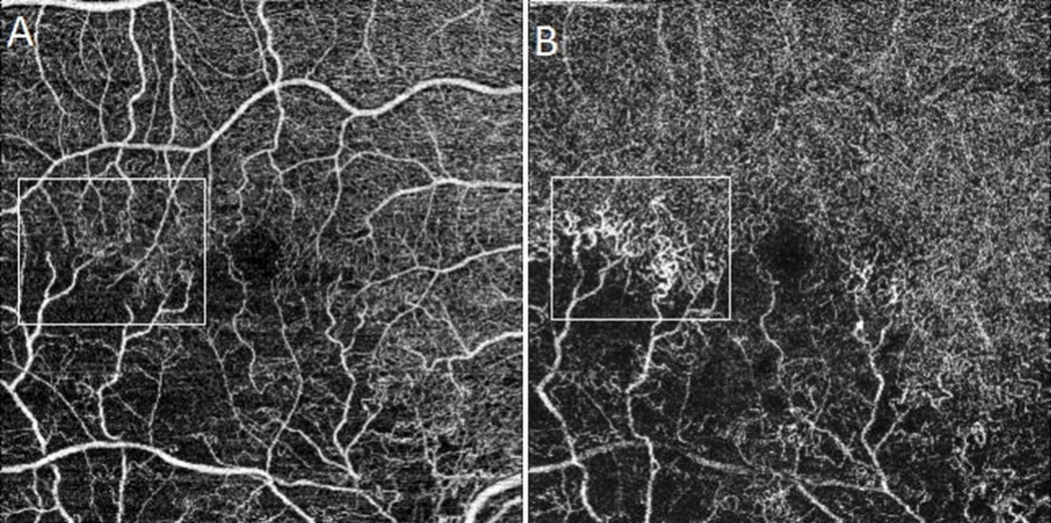
Image of a 60-year-old patient with BRVO. (**A**) Superficial capillary plexus; (**B**) Deep capillary plexus with retinal collaterals.

The variables derived from SSOCTA images were the foveal avascular zone (FAZ) and vascular parameters. FAZ was manually delineated by the same investigator on the superficial capillary plexus slab of the 6x6 mm SSOCTA scans using the free-hand tool of ImageJ (National Institute of Health, Bethesda, MD, USA), which can be seen in Fig 1. The presence of collateral vessels of the superficial (SCP) and deep capillary plexus (DCP) were subjectively analyzed in OCTA images (Fig 2). Collateral vessels were defined as vascular arcades or tortuous vascular networks in the retina [10]. SSOCTA images of the SCP were further analyzed with a semi-automated vessel analyzing software (AngioTool 64. Version 0.6a) [11]. The evaluation parameters were set for each image in order to assure optimal detection of the microvasculature. This program segments and skeletonizes blood vessels, and the morphometrical measurements of the vessels can be calculated [11]. As each side of the 6x6-mm image contained 320 pixels, each pixel was calculated to be 18.75μm. The descriptions of the variables detected by the AngioTool are summarized in Table 1 and Fig 1 shows the analyzed OCTA images [12].

**Table 1:** AngioTool variables and descriptions [11].

Variables	Description
**Vessel area (mm^2^)**	The area of the segmented vessels
**Vessel percentage area = VD (%)**	The area of the segmented vessels in percent
**Total number of junctions**	The total number of vessel junctions in the image
**Junction density**	The frequency of junctions
**Total vessel length (mm)**	The sum of Euclidean distances* between the pixels of all the vessels in the image
**Total number of endpoints**	The number of open-ended segments
**Mean lacunarity**	The mean lacunarity overall size boxes (lacunarity is a variable that indicates spatial dispersion)

*The Euclidean distance is the ordinary straight line distance between two points in Euclidean space, which can be any nonnegative integer dimension, including the three-dimensional space [12].

### SSOCT variables

Seven OCT scans were analyzed by the same investigator for each eye; one central B-scan and three B-scans above and below the foveal scan, following the method of a previous study from Sun et al [13]. The occurrence of intraretinal cysts (IRC), microaneurysm (MA), hyperreflective foci (HRF), epiretinal membrane (ERM), ellipsoid zone (EZ) and external limiting membrane (ELM) disruption, and disorganization of retinal inner layers (DRIL) were recorded.

**Fig 3:**
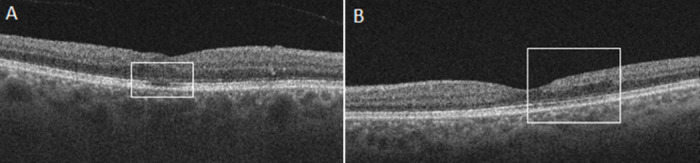
Example images of EZ disruption and DRIL. (**A)** Image of a 39-year-old patient with DR showing ellipsoid zone (EZ) disruption. (**B)** Image of a 43-year-old patient with RVO showing disorganization of retinal inner layers (DRIL) where five layers are affected.

DRIL was by definition determined when any boundaries between the ganglion cell- inner plexiform layer complex, inner nuclear layer, and outer plexiform layer could not be identified [13]. Intraretinal hypo-reflective round or elongated cystic structures were graded as IRC [14]. MAs were defined as ringed, round, or oval hyperreflective lesions in OCT images [15]. The presence of HRF was defined as small focal hyperreflective areas mainly in the outer retinal layers but they can occur in any retinal layers [16]. ERM was defined as a highly reflective layer on the retinal surface above the ILM layer [17]. Intact ELM and EZ were defined as a continuous hyperreflective line in the corresponding retinal layer [18]. The status of IRC, MA, HRF, ERM, EZ and ELM disruption were classified as absent or present when observed in at least one OCT scan. Fig 3 shows a typical example of EZ disruption and DRIL.

### Statistics

Statistical analysis was performed using SPSS V23 (IBM, Corp., Armonk, N.Y. USA). The data were summarized with descriptive statistics and displayed as the mean ± standard deviation (SD) or percent fraction of the total (%). First, we compared the subgroups of RVO (CRVO and BRVO) and DR (non proliferative diabetic retinopathy-NPDR and proliferative diabetic retinopathy-PDR) to evaluate if there were any differences in the distribution of the parameters. For further statistical analysis we pooled the subgroups together into a single RVO and DR group. SSOCT and SSOCTA variables were compared between DR and RVO eyes with the help of ANOVA for parametric and Mann-Whitney U-Test for non parametric measures. Dichotomous variables were compared using the Chi-square test. Correlations between the parameters were calculated with Spearman test, and between dichotomous variables with Point Biserial Correlation analysis. A p < 0.05 was considered the level of significance. Bonferroni correction was applied to account for multiple testing.

## Results

Forty-four eyes of 44 patients (17 female) were included, 22 with DR and 22 with RVO. Seven eyes of the RVO patients had CRVO (32%) and 15 had BRVO (68%). The diabetic group comprised 2 eyes with mild DR (9%), 3 eyes with moderate DR (13%), 4 eyes with severe DR (18%), and 13 eyes with proliferative/post proliferative DR (59%) according to the ETDRS scale. The mean patient age was 60.55 ± 11.38 years; mean decimal BCVA was 0.8 ± 0.36 (Snellen 20/25). All but 3 patients (7%) had received previous treatment because of their retinal condition, 75% because of macular edema and 24% because of proliferative DR. Thirty-three (75%) patients had received anti-VEGF (Aflibercept, Eylea, Bayer, Germany) treatment, but no injections at least two months before study inclusion. The mean number of anti-VEGF injections was 5.2 among patients with RVO and 2.0 among patients with DR. Thirteen DR patients (30%) had received laser photocoagulation, and 3 (6%) had undergone vitrectomy because of vitreous hemorrhage, tractional retinal detachment or epiretinal membrane. Five patients (11%) had undergone cataract surgery. A complete listing of the medical history is given in Table 2.

**Table 2:** Clinical characteristics of the patients.

Clinical characteristics	
**DR, n patient (%)**	22 (50)
**RVO, n patient (%)**	22 (50)
**Female, n patient (%)**	19 (43)
**Age, mean ± SD in years**	60.55 ± 11.38
**DR severity, n patient (% of DR patients)**	
**mild DR**	2 (9)
**moderate DR**	3 (13)
**severe DR**	4 (18)
**proliferative/postproliferative DR**	13 (60)
**RVO distribution, n patient (% of RVO patients)**	
**CRVO**	8 (36)
**BRVO**	14 (64)
**Visual acuity mean ± SD in Snellen decimal**	0.86 ± 0.35
**Lens status, n eyes (%)**	
**Phakic**	39 (89)
**Pseudophakic**	5 (11)
**Previous treatment, n patient (%)**	
**Anti-VEGF all patients**	33 (75)
**DR patients**	11 (50)
**RVO patients**	22 (100)
**Laser photocoagulation all patients**	13 (30)
**DR patients**	13 (59)
**RVO patients**	0
**Vitrectomy all patients**	3 (6)
**DR patients**	3 (14)
**RVO patients**	0

DR: diabetic retinopathy, RVO: retinal vein occlusion, CRVO: central retinal vein occlusion, BRVO: branch retinal vein occlusion, SD: standard deviation.

### Results of wide-field fluorescein angiography

We clarified peripheral retinal ischemia from wide-field fluorescein angiographies. From the twenty two RVO patients twenty had assessable wide-field angiographies. Six patients (30%) had no peripheral nonperfusion, while ≤5 disc diameter nonperfusion were observed in nine (40%) patients, and >5 disc diameter in five (25%) patients. Angiographies from DR patients showed that three (21%) patients had no peripheral nonperfusion. In eight patients (67%) ≤5 disc diameter and in two patients (14%) patients >5 disc diameter peripheral nonperfusion were observed. Twelve DR patients had no or not gradable angiographies. Based on this distribution, the two cohorts were comparable and the extent of peripheral ischemia probably did not affect the results.

### Subgroup analysis: Comparison of BRVO and CRVO, NPDR and PDR groups

The subgroups of RVO and DR were first analyzed. We compared the parameters of BRVO and CRVO patients and we found statistically significant difference only in one parameter: there were significantly more collateral vessels of the DCP (p = 0.014) in patients with BRVO. For further statistical comparison we pooled these two groups together into a single RVO group. The number of cases with mild (n = 2), moderate (n = 4) and severe (n = 3) DR were very low, so we decided to create two groups (NPDR and PDR) to evaluate if there were differences in the distribution of the parameters depending on the severity of the DR. We found no statistically significant differences between the two groups after Bonferroni correction.

### Comparison of DR and RVO groups

After subgroup analysis, we compared the pooled DR and RVO groups to determine if there were any statistically significant differences between them.

Significantly more MAs (p = 0.007) and ERM (p = 0.007) were found in patients with DR.

We found no statistically significant difference between DR and RVO groups for age (p = 0.55), for BCVA (p = 0.77), for FAZ (p>1), for DRIL (p = 0.54), for HRF (p = 0.37), for collateral vessels of SCP (p = 0.66), for DCP (p = 0.88), for EZ disruption (p = 0.73), for ELM disruption (p = 0.33) or vessel parameters (Table 3). The number of previous anti-VEGF injections was statistically significantly higher in RVO compared to the DR group (p< 0.001).

**Table 3:** Summary of the vessel variables with the p- values, showing the differences between DR and RVO groups.

Vascular characteristics	DR group Mean± SD	RVO group Mean± SD	All patients Mean± SD	P value
**Vessel area (mm^2^)**	10.27± 1.22	10.79± 1.62	10.52± 1.47	0.24
**Vessel density (%)**	28.8± 3.43	30.25± 4.51	29.52± 4.12	0.25
**Average vessel length (mm)**	5.21± 1.77	6.58± 2.64	5.89± 2.38	0.06
**Total number of junctions**	350.36 ± 52.28	354.4± 70.16	352.39± 62.62	0.83
**Junction density (%)**	9.83 ± 1.46	9.93± 1.95	9.88± 1.75	0.84
**Total vessel length (mm)**	2.73 ± 0.23	2.76± 0.31	2.75± 0.28	0.57
**Number of end points**	318.73 ± 32.1	310.36± 45.73	314.5± 40.2	0.72
**Mean lacunarity**	0.091± 0.02	0.097± 0.04	0.094± 0.35	0.88
**FAZ area (mm^2^)**	0.45± 0.39	0.45± 0.44	0.45± 0.42	0.68

FAZ: foveal avascular zone; SD: standard deviation; DR: diabetic retinopathy; RVO: retinal vein occlusion

### Descriptive statistics from all patients with central retinal ischemia

All the patients, regardless to the underlying disease, were pooled in one group to descriptively evaluate the distribution of OCT and OCTA parameters and correlate them to BCVA.

**Fig 4:**
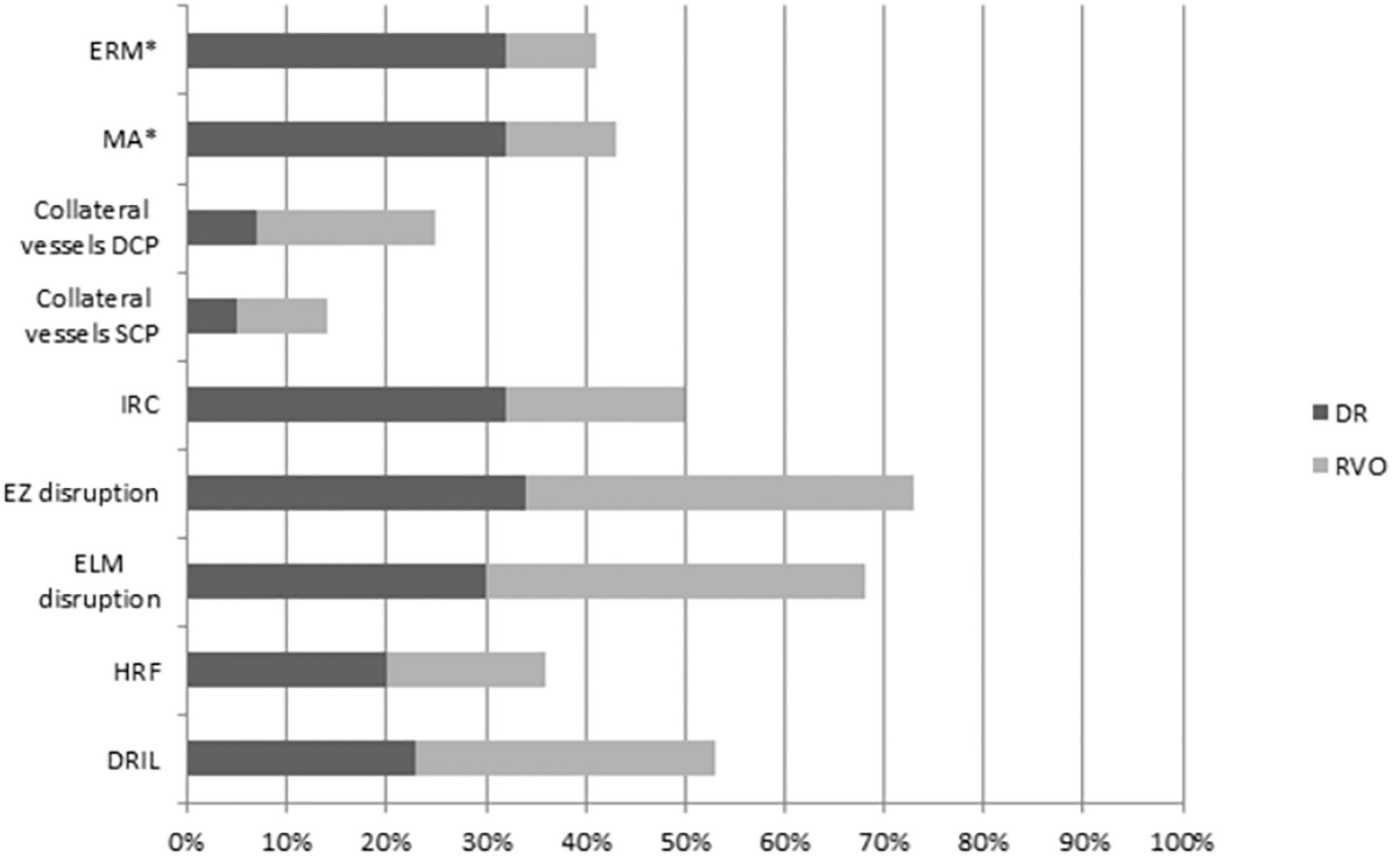
Distribution of OCT and OCTA features. Asterisk (*) indicates significant differences; (DRIL: disorganization of retinal inner layers; MA: microaneurysms; HRF: hyperreflective foci; ERM: epiretinal membrane; ELM: external limiting membrane; EZ: ellipsoid zone; SCP: superficial capillary plexus; DCP: deep capillary plexus).

The results of the descriptive statistic showed DRIL in 53%, HRF in 36%, ELM disruption in 68%, EZ disruption in 73% and IRC in 50% of all the patients with retinal ischemia. Collateral vessels of SCP were documented in 14% and of the DCP in 25% of all ischemic patients. 43% of all patients had MAs and 41% ERM. Fig 4 summarizes the distribution of OCT and OCTA features and the exact distribution of FAZ and vessel parameters are described in Table 3.

We further correlate OCT and OCTA features with BCVA to evaluate the relationship between morphological and functional parameters. We found a negative correlation between FAZ and BCVA (p = 0.045, r = -0.365). The correlation between BCVA and other parameters did not reach the statistically significant level (age: p = 0.063 r = -0.347, VD: p = 0.123 r = 0.31 and all other parameters p>1). FAZ and BCVA were also correlated for DR and RVO group separately. Both groups showed the same tendency: higher FAZ correlated with lower visual acuity. However, when we examined the two groups separately this value did not reach the statistically significant level (DR group: r = -0.341 p = 0.12, RVO group: r = -0.309, p = 0.16). These results are explained by the fact that the two groups are separately too small and summing up the two groups, the difference will be significant due to the larger simple size.

## Discussion

The aim of this study was to quantitatively assess OCT and OCTA parameters of patients with different entities of central retinal ischemia and to determine the correlation of the potential biomarkers with visual acuity.

Multiple studies have already investigated biomarkers and microvascular morphology of retinal ischemia [3, 4]. Quantifying vascular remodeling in retinal ischemic diseases has shown that specific OCT and OCTA variables, which are FAZ, DRIL length, VD, EZ disruption, central subfield thickness (CST) and intra retinal cysts are correlated with visual function. Therefore the functional outcome of both DR and RVO may be predictable [7, 19, 20].

We analyzed 44 eyes of 44 patients, 22 eyes with RVO and 22 eyes with DR and demonstrated that FAZ size is the best indicator for visual outcome in patients with macular nonperfusion. The comparison between the two disease groups showed significant difference only in the presence of MAs and ERM (significantly more in DR patients), the other vascular and morphological parameters did not differ significantly.

Previous studies showed that the size and morphology of FAZ is a reliable biomarker and the FAZ area is enlarged compared with healthy controls in both DR and RVO [5, 21]. In diabetic retinopathy, FAZ enlargement is one of the earliest signs that correlates significantly with visual acuity and DR severity [4, 7, 20]. Balaratnasingam et al. found a significant correlation between visual acuity and FAZ area in patients with DR and RVO, and showed that this relation is modulated by age [4]. Our results confirm the above-mentioned studies as the FAZ area was found to be significantly correlated with BCVA. It should be mentioned that this correlation did not reach the significant level when comparing FAZ and BCVA separately in DR and RVO group due to the small simple size. For further conclusions larger studies would be needed.

Another frequently analyzed parameter is vessel density, which has been found to be significantly decreased in both DR and RVO and correlated with visual acuity [5, 7]. Al-Sheikh et al. measured vessel density manually in healthy and diabetic eyes and found significantly lower vessel density in DR patients in both the SCP and DCP [7]. Kang et al. demonstrated FAZ enlargement, increased parafoveal capillary non-perfusion, and decreased parafoveal VD in eyes with RVO compared to healthy control subjects. These parameters were also found to be correlated with visual acuity [5]. In our study cohort the correlation between VD and BCVA did not reach the level of significance.

The vascular analyzing software, AngioTool, allows for quantitative assessment of morphological and spatial vessel parameters including the size of the vascular network, total and average vessel length, vascular density as well as the measurement of vessel lacunarity [11]. In the present study the vascular parameters were evaluated to investigate the morphological similarities and differences between central retinal ischemia caused by DR and RVO. After examining all the vascular parameters, we did not find statistically significant differences between the two groups which suggests that the OCTA-appearance of ischemia in the retinal microvasculature does not depend on the underlying disease.

Morphological OCT parameters, ELM and EZ disruption, HRF and DRIL have all been described as markers for visual outcome and ischemia in DR and RVO [3, 13, 22, 23]. In patients with existing or resolved center-involving diabetic edema, DRIL was found to be a prognostic marker of visual outcome. Patients with central DRIL show worse visual acuity, which explains why some patients have good vision despite persisting diabetic macular edema while others present with reduced vision under favorable therapy response [13]. Berry et al. analyzed RVO patients and demonstrated that DRIL was strongly correlated with baseline ischemic index and baseline FAZ size. The ischemic index (%) was calculated by measuring what percentage of the imaged pixels, which represented the fundus, were nonperfused [3].

DRIL provides information on the condition of the inner layers of the retina, and EZ and ERM disruption are suitable parameters for the outer retinal layers. The ellipsoid zone corresponds to the inner segment of the photoreceptors, so the status of the photoreceptors can be deduced from their integrity [21]. Kadomoto et al. analyzed RVO patients and showed that visual acuity was associated with the defect length of the foveal EZ band. Macular sensitivity assessed by microperimetry was found to be correlated with the length of EZ disruption [24]. Correlation between visual acuity and EZ disruption was also found in diabetic eyes [4]. The integrity of the ELM plays an important role in maintaining the retinal structure, metabolism, and homeostasis [21]. Alterations in Müller cells during different retinal pathologies may affect the ELM state and involve hyperreflective changes in the first outer OCT band [21]. In patients with RVO and DR, ELM disruption was found to be associated with the visual outcome [25].

Hyperreflective foci were described as being a very early subclinical barrier breakdown sign in DR and a prognostic factor for BCVA in RVO [23, 26].

In this study cohort we could not show any correlation between DRIL, HRF, ELM and EZ disruption and BCVA.

Collateral vessels in RVO develop because of the changed hemodynamic and increased hydrostatic pressure and appear as tortuous vessels in the retina or within the optic disc and are more frequently observed in eyes with ischemic BRVO [10, 27]. Our study could not show any significant differences in vascular collaterals between DR and RVO groups or correlation with BCVA. In the RVO group all collateral vessels of the DCP were found in BRVO patients. We demonstrated significantly more MAs and ERM in the diabetic cohort compared to RVO patients. These results confirm previous findings and suggest that MA is a diabetic-specific feature. Accordingly, ERM occurs more frequently in the context of DR especially when ALK has been performed [28, 29].

It should be noted that the distribution of severity in the diabetic group was unbalanced, with a predominance of PDR patients. Antaki et al. has recently demonstrated significant higher central and peripheral ischemia in diabetic patients with PDR compared with mild and moderate NPDR [30]. To avoid the effect of intergroup variability, we compared two subgroups of DR patients (NPDR, PDR). No significant differences were found in the analyzed parameters. These results can be explained by the fact that patients were selected on the basis of the degree of ischemia, so they had similar vascular parameters despite of different DR severity.

Disease duration was much longer in the diabetic group, which might be caused by the different nature and progression of the diseases.

The effect of anti-VEGF treatment on the retinal vasculature has been analyzed in a number of studies and most of them found no differences in parafoveal vascular parameters before and after anti-VEGF therapy [31, 32]. Two studies found significant changes after anti-VEGF injections, but also demonstrated that those effects were transient and approximately 7 weeks after the treatment they were no longer detectable [32, 33]. In order to avoid potential effects of the drug on blood vessels, only those patients were included who had not received an injection at least 8 weeks before study inclusion.

For the evaluation of retinal function in central ischemia, we assessed BCVA. Previous studies used different approaches including retinal sensitivity as assessed by microperimetry. Retinal sensitivity were found to be significantly lower in ischemic areas [24]. A correlation was found between retinal sensitivity and disease duration and EZ disruption in both DR and RVO [24, 34, 35]. The strong association between these variables indicates that as soon as ischemia is present, the functional outcome is similar regardless of the original cause of the ischemia.

Some limitations of this study have to be considered including its retrospective nature and the relatively small sample size. Another important limitation is that patients were grouped together regardless of the occluded vascular segment and diabetic retinopathy severity, which may affect our results despite the fact that no differences were found between the subgroups. It should also be mentioned that the OCTA quality of the deep capillary plexus was not satisfactory for the quantitative evaluation of the vascular parameters. Despite these limitations our study has great importance because easily acquirable OCT and OCTA parameters can help clinicians predict disease prognosis in center involving retinal ischemia.

In conclusion, our study shows that FAZ is the most reliable biomarker for predicting the visual outcome in patients with central retinal non-perfusion. We did not find any significant OCT or OCTA based morphological and vascular differences between DR and RVO groups apart from the presence of disease specific features such as microaneurysms and epiretinal membrane. These results support the assumption that OCTA-appearance of ischemia in the retinal microvasculature is not dependent on the underlying disease and have similar outcomes as soon as ischemia is present. In order to fully confirm these assumptions, larger and prospective studies with higher resolution of both retinal vascular plexus are needed.
